# Geometrical Description in Binary Composites and Spectral Density Representation

**DOI:** 10.3390/ma3010585

**Published:** 2010-01-21

**Authors:** Enis Tuncer

**Affiliations:** Applied Superconductivity Group, Fusion Energy Division, Oak Ridge National Laboratory, Oak Ridge TN 37831-6122, USA; E-Mail: tuncere@ornl.gov; Tel.: +1-865-574 0705; Fax: +1-865-574 6122

**Keywords:** composite materials, binary mixtures, dielectric permittivity, spectral density representation, fractal geometry, **PACS** 77.22.-d Dielectric properties of solids and liquids, 78.20.-e Optical properties of bulk materials and thin films, 77.22.Ch Permittivity (dielectric function), 77.84.Lf Composite materials, 02.70.Hm Spectral methods, 02.70.Uu Applications of Monte Carlo methods, 05.45.Df Fractals, 07.05.Kf Data analysis: algorithms and implementation, data management, 61.43.-j Disordered solids

## Abstract

In this review, the dielectric permittivity of dielectric mixtures is discussed in view of the spectral density representation method. A distinct representation is derived for predicting the dielectric properties, permittivities *ε*, of mixtures. The presentation of the dielectric properties is based on a scaled permittivity approach, $\xi =\left(\varepsilon_{e}-\varepsilon_{m}\right){\left(\varepsilon_{i}-\varepsilon_{m}\right)}^{-1}$, where the subscripts e, m and i denote the dielectric permittivities of the effective, matrix and inclusion media, respectively [Tuncer, E. *J. Phys.: Condens. Matter* **2005**, *17*, L125]. This novel representation transforms the spectral density formalism to a form similar to the distribution of relaxation times method of dielectric relaxation. Consequently, I propose that any dielectric relaxation formula, *i.e.,* the Havriliak-Negami empirical dielectric relaxation expression, can be adopted as a scaled permittivity. The presented scaled permittivity representation has potential to be improved and implemented into the existing data analyzing routines for dielectric relaxation; however, the information to extract would be the topological/morphological description in mixtures. To arrive at the description, one needs to know the dielectric properties of the constituents and the composite prior to the spectral analysis. To illustrate the strength of the representation and confirm the proposed hypothesis, the Landau-Lifshitz/Looyenga (LLL) [Looyenga, H. *Physica* **1965**, *31*, 401] expression is selected. The structural information of a mixture obeying LLL is extracted for different volume fractions of phases. Both an in-house computational tool based on the Monte Carlo method to solve inverse integral transforms and the proposed empirical scaled permittivity expression are employed to estimate the spectral density function of the LLL expression. The estimated spectral functions for mixtures with different inclusion concentration compositions show similarities; they are composed of a couple of bell-shaped distributions, with coinciding peak locations but different heights. It is speculated that the coincidence in the peak locations is an absolute illustration of the self-similar fractal nature of the mixture topology (structure) created with the LLL expression. Consequently, the spectra are not altered significantly with increased filler concentration level—they exhibit a self-similar spectral density function for different concentration levels. Last but not least, the estimated percolation strengths also confirm the fractal nature of the systems characterized by the LLL mixture expression. It is concluded that the LLL expression is suitable for complex composite systems that have hierarchical order in their structure. These observations confirm the finding in the literature.

## 1. Introduction

Electrical properties of composite materials have attracted researchers to seek relationships between overall composite properties and intrinsic properties of the parts forming the mixture (constituents) and their spatial arrangement in the mixture [1,2,3,4,5,6,7,8,9,10,11,12,13,14]. Mixture formulas based on analytical and effective medium approaches were developed such that for various arrangements of inclusions, predicting the dielectric properties of composites was plausible [15,16,17,18].

A deep understanding of dielectric mixtures would be of great value for (i) calculating the dielectric constant of a mixture composed of substances with known dielectric constants, (ii) calculating the dielectric constant of the second component of a two-component mixture when the dielectric constants of the mixture and the first component are known [1], and (iii) estimating the morphology of a two-component mixture when the dielectric constants of the mixture and each of the components are known [19,20,21].

During the 1970s Fuchs [22,23] illustrated that the dielectric permittivity of mixtures could be expressed as a summation of depolarization factors. This approach took into account that each particle would yield a different depolarization factor depending on its shape. The beauty was that the summation term would yield the concentration of the individual shapes. In the late 1970s, Bergman showed that one can separate the geometrical contributions from the pure dielectric response of a composite if and only if the dielectric properties of the constituents are known [24,25,26]. Milton corrected errors in Bergman’s original derivation [27,28,29], and later Golden and Papanicolaou [30,31] gave the rigorous derivation for the spectral representation theory. If the Fuchs and Bergman approaches are studied in detail, both correspond to the same formalism, which is spectral density representation [19,20,23,24,32,33,34].

Notice that the present author has illustrated similarities between the dielectric relaxation and dielectric response of dielectric mixtures using the spectral density representation; the origin of similarities is very significant for the comprehension of the physics of dielectrics [21].

The concept of having cognition about the structure of composites, how the phases are arranged, is very useful in materials design and characterization, because special materials can be manufactured with a knowledge of structure-property relationships. We should not forget that the functionality in nature is created with structure. For regular arrangements of phases, there exist equations based on theoretical calculations of simple enough geometries [15,18]. However, disordered and fractal structures are abundant in nature [35,36], and a comprehension of materials properties with disordered or fractal structures has been a challenge for researchers for some decades. The fractal geometry or systems indicating hierarchical order has been one of the interesting topics in applied and theoretical (mathematical) physics [35,37,38,39,40]. As an example, the electrical properties of metal aggregates in insulating matrix media were studied extensively [36,41,42,43,44,45,46,47,48,49]. In these studies either the dimension of the electrical network or system, or the resonance frequency of the electrical impedance was used as a measure to indicate the fractal dimensions.

When real systems are taken into account, the structural information is, on the other hand, usually obtained by optical/microscopic techniques, this information is later analyzed to estimate the fractal dimensions. While if one applies the spectral density approximation to electrical data of a mixture, structural information could be obtained—whether the mixture has regular, disordered or fractal structural characteristics or not. We should consider the application of the spectral density to the mixture immittance (Immittance term is used to express both impedance and admittance in a combined from.) data as optical or X-ray techniques such that structural characteristics of the mixture are determined with the analysis—the resolved depolarization factors corresponds to the arrangements of phases [19,20,49].

One can also utilize a dielectric mixture formula to model the electrical properties of the composite system in hand, such that the model contains structural information, e.g., there exist effective medium theories for composites with spherical and ellipsoidal inclusions [3,50,51]. In this review, we employ the spectral density representation as the general representation approach for composites to resolve the geometrical description of a model system described by the Landau-Lifshitz/Looyenga (LLL) effective medium formula [52,53], or in other words we challenge the physical significance of the LLL expression.

The LLL expression was extensively used to describe the dielectric properties of dispersive systems composed of powders or exhibiting porous structure [3,54,55,56,57,58,59,60,61,62,63,64,65,66,67]. It was even shown [55] that the LLL formula was more reliable when mixtures contained strongly dissipative particles and was compared to others like Maxwell Garnett (MG) [68,69], Bruggeman [70], *etc.* (see for example Refs. [1,2,3,4,5,6,7,8,9,10,11,12] for other formulas). First, it is presented that the spectral density representation can in fact be written in a novel, elegant form that can be implemented in already existing dielectric data analysis techniques [71,72,73,74,75]. Later, the simplified notation is used with a numerical procedure which is derived to solve inverse problems [19,20,76,77] to resolve the spectral densities in the LLL [52,53] dielectric mixture expression. The significance of the presented approach on dielectric mixtures is also discussed.

The paper is organized as follows. The spectral density representation for a binary mixture is presented in §2. In addition, similarities between dielectric relaxation in dielectrics and dielectric permittivity of binary mixtures are illustrated. The dielectric data representation is described in §3., and hints are given for analyzing impedance data of mixtures. The numerical method for solving the inverse integral is also presented explicitly for interested readers in §4. The numerical data generation and the LLL expression are presented in §5. The results obtained by the inverse integral solution and by the proposed conventional dielectric dispersion expression are compared in §6. The conclusions are in contained §7.

## 2. Spectral Density Representation

In spectral density representation analysis of binary mixtures, the dielectric permittivity of a heterogeneous (effective) medium is expressed as [24,25,26,27,28,29,30,31,33,78,79]

(1) $$\begin{array}{c} \varepsilon_{e}=\varepsilon_{m}\left\{1+q\left[A\left(\frac{\varepsilon_{i}}{\varepsilon_{m}}-1\right)+\int_{0}^{1}\frac{G(x)dx}{{\left(\frac{\varepsilon_{i}}{\varepsilon_{m}}-1\right)}^{-1}+x}\right]\right\} \end{array}$$

where $\varepsilon_{e}$, $\varepsilon_{m}$ and $\varepsilon_{i}$ are the complex dielectric permittivity of the effective, matrix and inclusion media, respectively; *q* and *x* are the concentration of inclusions and the spectral parameter, respectively. The function G(x) is the spectral density function (SDF) and possesses information about the topological description of the mixture. Eq. (1) can be arranged in a simplified form, cf. § 7, as follows:

(2) $$\begin{array}{c} \xi =\xi_{s}+q\int_{0}^{1}\frac{G(x)dx}{1+\varepsilon_{m}^{-1}\Delta_{\mathrm{im}}x} \end{array}$$

where $\Delta_{\mathrm{im}}=\varepsilon_{i}-\varepsilon_{m}$ and *ξ* is the complex and frequency dependent “scaled” permittivity [21]

(3) $$\begin{array}{c} \xi =\frac{\varepsilon_{e}-\varepsilon_{m}}{\varepsilon_{i}-\varepsilon_{m}} \end{array}$$

The constant $\xi_{s}$ in Equation 2 is complex and depends on the concentration and structure of the composite; its real part is related to the so-called “percolation strength” [33,80]. The mathematical properties and conditions that SDF satisfies are presented in §7. [26,33,78,79,81].

Equation (3) is a very similar expression to the distribution of relaxation times (DRT) representation of a broad dielectric dispersion (relaxation) [21,76,82,83,84,85,,86,87,88,89]

(4) $$\begin{array}{c} \varepsilon \left(ı\omega \right)=\varepsilon_{\infty }+\Delta \varepsilon \int_{0}^{\infty }\frac{G(\tau )d\tau }{1+ı\omega \tau } \end{array}$$

where *ε*, $\varepsilon_{\infty }$ and Δε are the complex permittivity, permittivity at optical frequencies and dielectric strength, respectively; and $ı\equiv \sqrt{-1}$. The quantities *ω* and *τ* are the angular frequency and relaxation time, respectively. The distribution function for the relaxation times is G(τ). Comparison of Equations (2) and (4) demonstrates that both the DRT and the scaled permittivity of SDF are actually the same. However, the new complex parameter *ϖ* in SDF $\left(\varpi \equiv \varepsilon_{m}^{-1}\Delta_{\mathrm{im}}\right)$ is similar to the pure complex frequency ıω in DRT representation, and the real number constant $\varepsilon_{\infty }$ is a complex number $\xi_{s}$ in SDF representation.

The significance of $\xi_{s}$ is that, just like $\varepsilon_{\infty }$, it does not indicate any dispersion in the dielectric spectra, meaning that it does not contribute to the dielectric relaxation. One can also look at these two parameters as regions with no charge or dipolar movements. In addition, the spectral parameter *x* is similar to the relaxation time *τ* in the DRT. Finally, the dielectric strength Δε in the DRT representation is like the concentration of inclusions in the SDF.

Because of these similarities between the SDR and DRT approaches, methods developed for dielectric data analysis [71,73,74,75] can be applied to the scaled permittivity *ξ* of SDF [19,20,21,34,49]. For example, one of the most employed dielectric dispersion expressions, the Havriliak-Negami empirical expression [90], can be used to analyze the scaled complex dielectric permittivity data of a mixture [34],

(5) $$\begin{array}{c} \xi \left(\varpi \right)=\xi_{s}+\frac{q}{{\left[1+{\left(\varpi x\right)}^{\alpha }\right]}^{\beta }} \end{array}$$

where *α* and *β* are parameters of a general distribution function [73,90], and *ϖ* is the scaled complex frequency. When α=β=1, a Debye-type relaxation is observed in the dielectric dispersion representation [91]. In the case of spectral density representation, however, the Maxwell Garnett approximation is obtained for α=β=1 and x=(1-q)/d, since G(x)=δ[x-(1-q)/d] [19,20,21,78,79,81], where *d* is the dimensionality of the system.

One can, therefore, in principle write a new, more general empirical mixture formula [34] by isolating the dielectric permittivity $\varepsilon_{e}$ of the composite in Equation (3) and substituting it in Equation (5),

(6) $$\begin{array}{c} \varepsilon_{e}=\varepsilon_{m}+\Delta_{\mathrm{im}}\left\{\xi_{s}+\frac{q}{{\left[1+{\left(\varpi x\right)}^{\alpha }\right]}^{\beta }}\right\} \end{array}$$

Finally, note that the fractional expression inside the curly parentheses in Equation (6) can be exchanged with any of the dielectric dispersion relations in the literature [71,74,90,92,93,94,95,96].

## 3. Representation of Dielectric Data

The dielectric function for a *d*-dimensional (or composite with arbitrarily shaped inclusions) is defined as follows with Maxwell Garnett (MG) expression for a composite [68,69]

(7) $$\begin{array}{c} \varepsilon_{e}^{\mathrm{MG}}\left(\omega ;\;\varepsilon_{m},\;\varepsilon_{i},\;q,\;d\right)=\varepsilon_{m}+\frac{\varepsilon_{m}\;d\;q\;\Delta_{\mathrm{im}}}{\left(1-q\right)\;\Delta_{\mathrm{im}}+d\;\varepsilon_{m}} \end{array}$$

**Figure 1:**
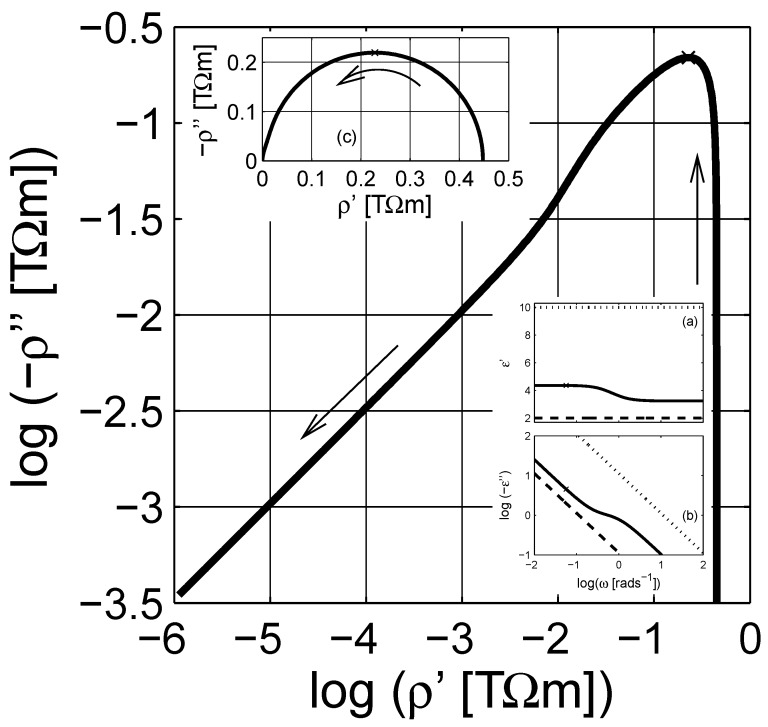
The Argand diagram of resistivity for a Maxwell Garnett composite in log-log scale, the effective permittivity is calculated with $\varepsilon_{e}=\varepsilon_{e}^{\mathrm{MG}}\left(\omega ;\varepsilon_{m},\;\varepsilon_{i},\;0.3,3\right)$, with $\varepsilon_{m}=2+10^{-12}{\left(ı\varepsilon_{0}\omega \right)}^{-1}$ and $\varepsilon_{i}=10+10^{-10}{\left(ı\varepsilon_{0}\omega \right)}^{-1}$. The arrows indicate the direction of increasing angular frequency *ω*. Insets (a) and (b) are the real and the imaginary parts of permittivities; $\varepsilon_{e}$, $\varepsilon_{m}$ and $\varepsilon_{i}$ are the permittivities for the matrix (– – –), inclusion (- - - - -) and effective (——–) media. Inset (c) is the Argand diagram of resistivity in linear-linear scale. Logarithmic scale is base 10.

The dielectric data of the composite can be expressed in one of the four immittance representations [72,74,83]: (*i*) the complex resistivity ρ(ω), (*ii*) the complex modulus $M\left(\omega \right)\equiv ı\omega \varepsilon_{0}\rho \left(\omega \right)$; (*iii*) the complex permittivity $\varepsilon \equiv {\left[M\left(\omega \right)\right]}^{-1}$, and (*iv*) the complex conductivity $\sigma \left(\omega \right)\equiv ı\omega \varepsilon_{0}\varepsilon \left(\omega \right)\equiv {\left[\rho \left(\omega \right)\right]}^{-1}$. When we are dealing with frequency dependent dielectric properties of composites, the effective conductivity of the composite can sometimes influence the imaginary part of the dielectric function—hindering the dielectric losses due to the interfacial polarization—as shown in inset (b) of Figure 1. In such cases it is more appropriate to use the complex resistivity representation (plot) as shown in the Argand diagrams in log-log and linear scales in Figure 1 and 1c, respectively.

**Figure 2:**
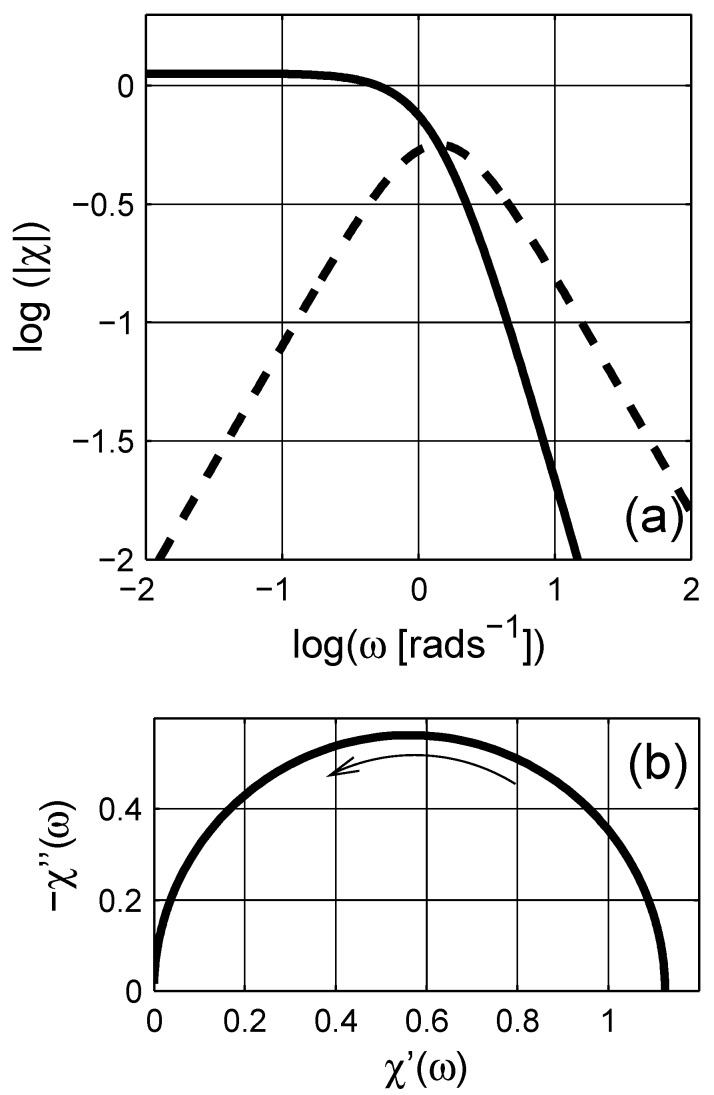
(a) Dielectric susceptibility *χ* as a function of angular frequency *ω*; the real and the imaginary parts are presented with solid (——–) and dashed (– – –) lines, respectively. The arrow indicates the direction of increasing angular frequency *ω*. (b) The Argand diagram (Cole-Cole plot) of susceptibility. Logarithmic scale is base 10.

The ohmic conductivity $\sigma_{e,dc}$ (or resistivity $\rho_{e,dc}$) of the material can be estimated from the complex resistivity Argand plot. However, once the conductivity contributions are cleared from the immittance or dielectric data, using the estimated resistivity value in Figure 1 as ω→0, the pure dielectric dispersion (permittivity) would be obtained. In addition, the high frequency dielectric permittivity $\varepsilon \left(\omega \to \infty \right)\equiv \varepsilon_{\infty }$ can be further subtracted from the data to obtain the pure dielectric polarization (susceptibility *χ*) of the composite as presented in Figure 2; $\chi =\chi^{\prime }-ı\chi^{\prime \prime }\equiv \varepsilon -\varepsilon_{\infty }+ı\sigma_{dc}{\left(\varepsilon_{0}\omega \right)}^{-1}$. The imaginary part of *χ* has a peak around $\omega ∼1^{+}\;\mathrm{rad}s^{-1}$. The linear scale plot of the susceptibility *χ* is a semicircular curve, cf. Figure 2b, as seen in Figure 1c.

**Figure 3:**
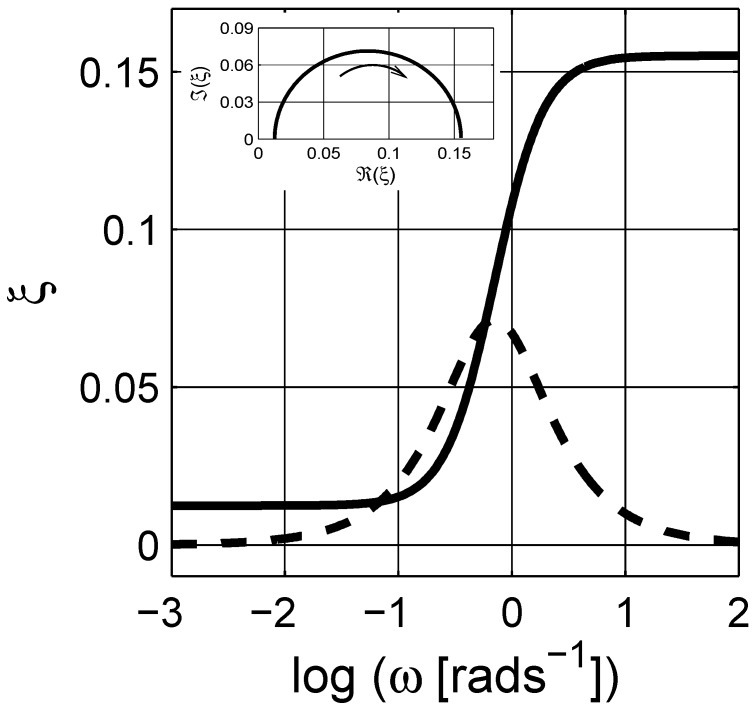
The real (ℜ(ξ); ——–) and the imaginary (ℑ(ξ); – – –) parts of scaled permittivity *ξ* in the Maxwell Garnett approximation. Inset is the Argand diagram for *ξ* with the arrow showing the direction of increasing angular frequency *ω*. The inclusions are spherical d=3, and q=0.3. Logarithmic scale is base 10.

If we now consider the frequency dependent properties of the scaled permittivity *ξ* for the considered MG composite, the real part and the imaginary parts are similar to the dielectric permittivity $\varepsilon_{e}$ of the composite, cf. inset in Figure 3, which shows the Argand diagram of the scaled permittivity *ξ*; observe that the increasing frequency is in the opposite direction when compared to the Argand plot of the susceptibility in Figure 2b. The real part of *ξ* is a mirror image of $ℜ(\varepsilon_{e})$. Unlike the imaginary part of $\varepsilon_{e}$ (due to ohmic losses), ℑ(ξ) shows a clear peak around $\omega ∼1^{-}\;\mathrm{rad}s^{-1}$. The shift in the origin position in the Argand diagram in the inset of Figure 3 is related to the percolation strength $\xi_{s}$, which is close to zero, and the concentration of the inclusions.

**Figure 4:**
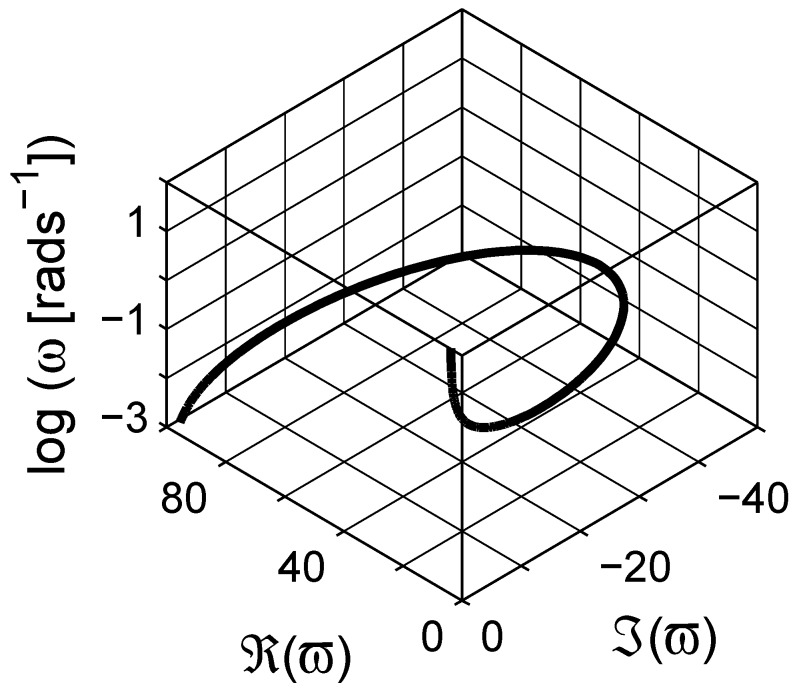
Dependence of the real frequency *ω* as a function of complex “scaled” frequency *ϖ* in the spectral representation. Logarithmic scale is base 10.

**Figure 5:**
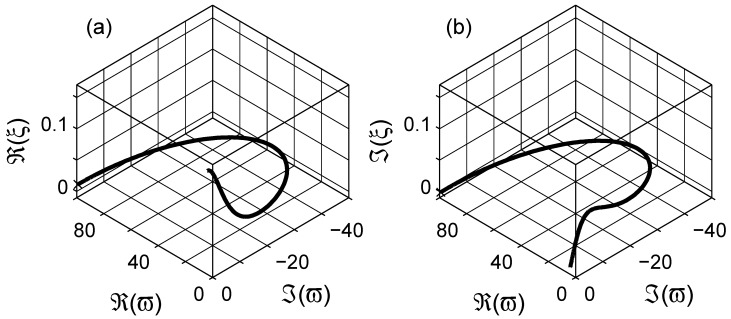
Three-dimensional line plots of (a) the real ℜ(ξ) and (b) the imaginary ℑ(ξ) parts of the “scaled” permittivity for a Maxwell Garnett mixture. The inclusions are spherical d=3, and q=0.3.

There are similarities between the susceptibility and the scaled permittivity plots when the same frequency *ω* axis is used; however, note that the scaled frequency *ϖ* for the scaled permittivity is a complex quantity. We therefore illustrate the dependence of the real angular frequency *ω* as a function of *ϖ* in a three dimensional curve-plot in Figure 4. In addition, the real and the imaginary parts of the scaled permittivity *ξ* are shown in Figure 5 as a function of *ϖ*. As shown in the figure, the actual dependence of *ξ* on *ϖ* is more complicated than *ε* on *ω*. On the contrary, this dependence can be used to estimate the spectral density function for a given system, which is explicitly given with a numerical procedure in the next section.

## 4. Numerical Estimation of Spectral Density Function

The derived spectral density expression in Equation 2 is a Bolter equation [97], which is a special form of the Fredholm integral equations [98]. Such equations are usually considered to be *ill-conditioned* because of their non-unique solutions. However, the approach used here and presented several times elsewhere [19,73,76,77,89] leads to unique solutions by means of a constrained least-squares fit and the Monte Carlo integration methods. Some other approaches to solving the spectral density function are also suggested in the literature [33,78,79,80,81,99,100,101,102]. The presented numerical method to solve inverse integral transforms has been previously previously been used in different problems [19,20,72,73,76,77,89]. In this particular approach, the integral in Equation (3) is first written in a summation form over some number of randomly selected and fixed $x_{n}$-values, $x_{n}\in \left[0,1\right]$, where *n* is less than the total number *M* of experimental (known) data points in the complex scaled permittivity, *ξ*,

(8) $$\begin{array}{c} \xi =\xi_{s}+\sum \frac{g_{n}}{1+\varpi x_{n}}\;n\leq M \end{array}$$

This converts the non-linear problem in hand to a linear one with $g_{n}$ being the unknowns, weights of the randomly selected $x_{n}$ values. In the present notation g is qG. Later, a constrained least-squares algorithm is applied to get the corresponding g-values and $\xi_{s}$,

(9) $$\begin{array}{c} \min\sum {\left[\underline{\xi }-Kg\right]}^{2}\;\mathrm{and}\;g\geq 0 \end{array}$$

where K is the kernel-matrix,

(10) $$\begin{array}{c} K=\left(\begin{array}{cccc} 1 & K_{11} & K_{12} & \ldots \\ 1 & K_{21} & K_{22} & \ldots \\ 1 & K_{31} & K_{32} & \ldots \\ \vdots & \vdots & \vdots & \ddots \end{array}\right) \end{array}$$

Here $K_{ij}={\left[1+\varpi_{i}x_{j}\right]}^{-1}$; index *i* runs on the angular frequency points i=1,⋯,M; and index *j* runs on the randomly selected *x* values, j=1,⋯,n. The parameters $\underline{\xi }$ and g in Equation (9) are column vectors, respectively, the scaled permittivity calculated from the experimental (known) data and the searched spectral density,

(11) $$\begin{array}{ccc} \underline{\xi }=\left(\begin{array}{c} \xi [\varpi \left(\omega_{1}\right)]\\ \xi [\varpi \left(\omega_{2}\right)]\\ \xi [\varpi \left(\omega_{3}\right)]\\ \xi [\varpi \left(\omega_{4}\right)]\\ \vdots \end{array}\right) & \;\mathrm{and}\; & g=\left(\begin{array}{c} \xi_{s}\\ g_{1}\\ g_{2}\\ g_{3}\\ \vdots \end{array}\right) \end{array}$$

In our numerical procedure, we perform many minimization steps with fresh, new sets of randomly selected $x_{j}$-values. Here we have adopted $2^{12}$ minimization steps. The $g_{j}$-values and $\xi_{s}$ obtained are recorded in each step, which later build up the spectral density distribution g and a distribution for the percolation strength, $\xi_{s}$. For a large number of minimization loops, the *x*-axis actually becomes continuous—the Monte Carlo integration hypothesis—contrary to regularization methods [80,101]. The number *M* of data points chosen is 24, and the number *N* of unknown g-values is 22. In the analysis presented below, the total number of randomly selected *x* values is $2^{12}\times 22=90112$.

Application of the numerical procedure to the Maxwell Garnett expression, impedance data of a porous rock-brine mixture and two-dimensional “ideal” structures, and the accuracy in the numerical inversion, have previously been presented elsewhere [19,20]. The estimated spectral density functions for the MG expression were delta sequences [103] as expected, without any significant percolation component, because of the estimated concentration *q*, cf. Equation (20). In the next section, we apply the numerical procedure to the Landau-Lifshitz/Looyenga [52,53] expression to better understand the nature of dielectric mixtures, which obey this relation.

## 5. Landau-Lifshitz/Looyenga Expression

Landau and Lifshitz [52] and Looyenga [53] independently, using different approaches, developed an expression for dielectric mixtures, that implies the additivity of cube roots of the permittivities of mixture constituents when taken in proportion to their volume fractions (see Refs. [3,54,55,56,57,58,59,60,61,62,63,64,65,66,67] for examples).

(12) $$\begin{array}{c} {\left[\varepsilon_{e}^{\mathrm{LLL}}\left(\omega ;\varepsilon_{m},\;\varepsilon_{i},\;q\right)\right]}^{1/3}=\left(1-q\right)\;\varepsilon_{m}^{1/3}+q\;\varepsilon_{i}^{1/3} \end{array}$$

This expression is used extensively in the literature for powdered materials and optical properties of material mixtures [3,56]. In the following calculations, we choose the same values for the dielectric functions of the phases as before, cf. Figure 1. The concentration *q* of the inclusion phase is varied between 0.1 and 0.9 in the simulations.

The extracted spectral functions are like distributions, and they are analyzed by means of comparing them with a known distribution. We apply the Lévy statistics [104,105,106,107], which are widely used for interacting systems in different research fields [19,89,106,107,108,109,110,111,112]. The Lévy stable distribution is a natural generalization (approximation) of the normal (Gaussian), Cauchy or Lorenz and Gamma distributions. It is used when analyzing sums of independent, identically distributed, random variables by a diverging variance. Its characteristic function is expressed as

(13) $$\begin{array}{c} L\left(x;A,\mu ,\gamma ,\zeta \right)=A|\exp\{-|\zeta \left(x-\mu \right){|}^{\gamma }\}| \end{array}$$

Here, *γ* is the characteristic exponent (γ>0), *μ* is the localization parameter, *ζ* is the scale parameter and A is the amplitude. The special forms of Equation (13) are the Gaussian [L(x;A,μ,2,ζ)], the Lorentz or Cauchy [L(x;A,μ,1,ζ)] and Gamma [L(x;A,μ,1/2,ζ)] distributions. Different forms of probability density functions for Lévy statistics exist, and we adopted a stable distribution used in the literature [104,105,106]. We omitted the imaginary parts in the characteristic function because of their insignificance in the results.

**Figure 6:**
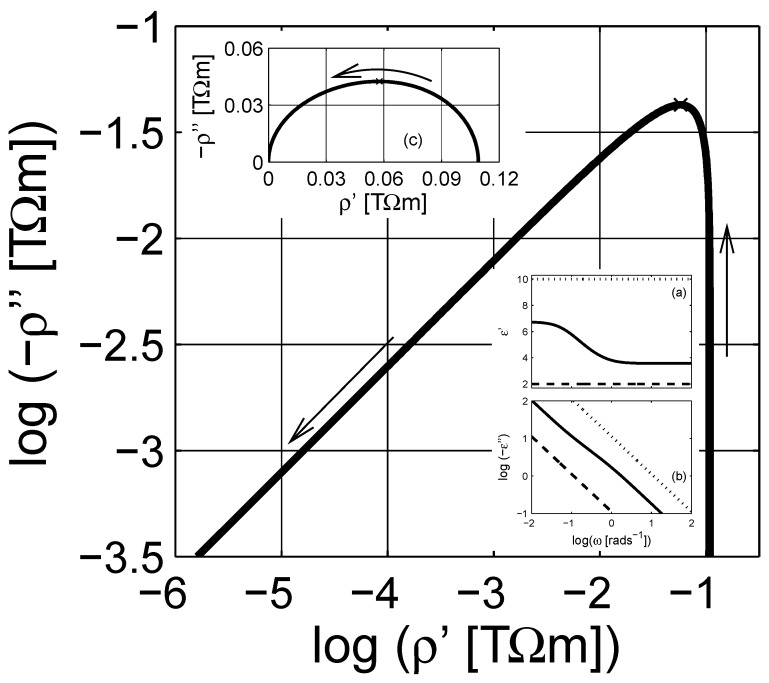
The Argand diagram of resistivity for a Landau-Lifshitz/Looyenga composite in log-log scale; the effective permittivity is calculated by $\varepsilon_{e}=\varepsilon_{e}^{\mathrm{LLL}}\left(\omega ;\varepsilon_{m},\;\varepsilon_{i},\;0.3,\;3\right)$, with $\varepsilon_{m}=2+10^{-12}{\left(ı\varepsilon_{0}\omega \right)}^{-1}$ and $\varepsilon_{i}=10+10^{-10}{\left(ı\varepsilon_{0}\omega \right)}^{-1}$. The arrows indicate the direction of increasing angular frequency *ω*. Insets (a) and (b) are the real and the imaginary parts of permittivities; $\varepsilon_{e}$, $\varepsilon_{m}$ and $\varepsilon_{i}$ are the permittivities for the matrix (– – –), inclusion (- - - - -) and effective (——–) media. Inset (c) is the Argand diagram of resistivity in linear-linear scale. Logarithmic scale is base 10.

**Figure 7:**
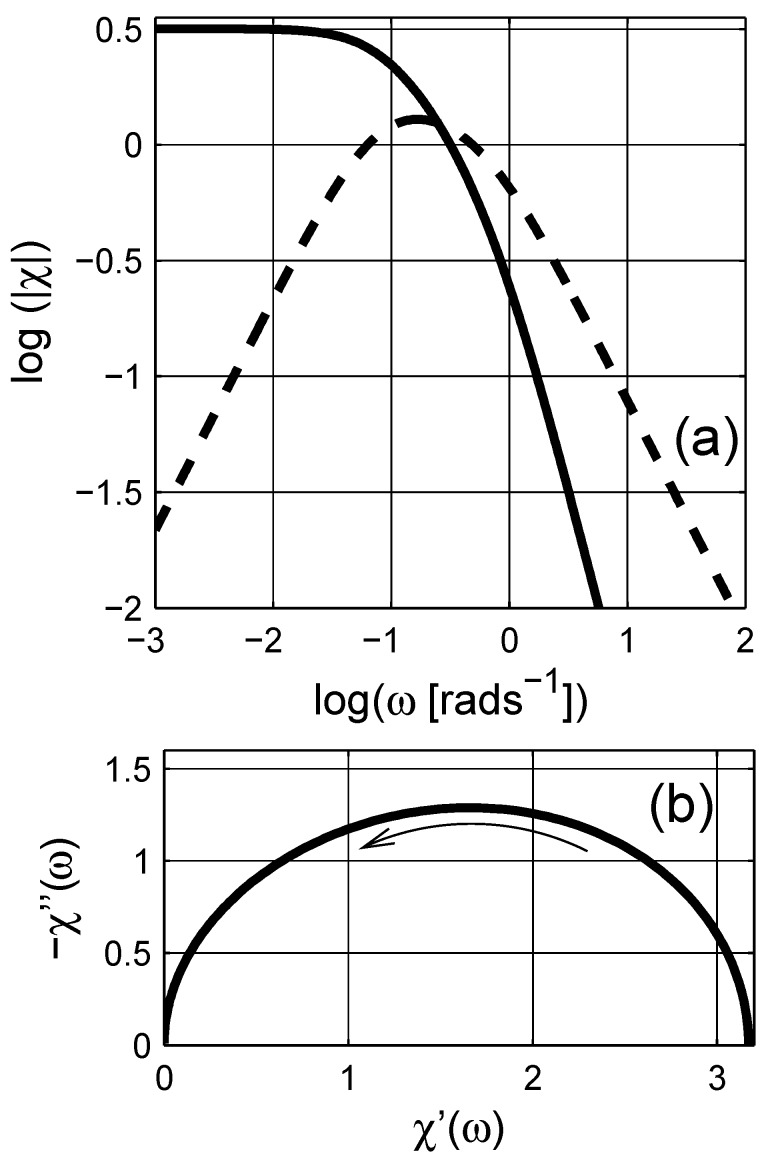
(a) Dielectric susceptibility *χ* as a function of angular frequency *ω*; the real and the imaginary parts are presented with the solid (——–) and dashed (– – –) lines, respectively. The arrow indicates the direction of increasing angular frequency *ω*. (b) The Argand diagram (Cole-Cole plot) of susceptibility. Logarithmic scale is base 10.

In Figure 6, the simulated dielectric permittivity with Equation (12) is presented at the complex resistivity level. The actual dielectric data is shown in the insets in Figure 6a,b. Compared to the MG expression in Equation 7, shown in Figure 1, the LLL expression does not show a knee-point as the complex resistivity decreases with increasing frequency. In addition, the linear scale Argand diagram of the LLL expression in Figure 6c is not a perfect semicircle as the MG one. The dielectric susceptibility after subtraction of the frequency independent parameters $\varepsilon_{\infty }$ and *σ* is presented in Figure 7. The losses, $\chi^{\prime \prime }$, are non-symmetrical for the LLL expression, which indicates that the actual dielectric response can be modeled by a non-Debye dielectric dispersion, e.g., the Havriliak-Negami expression; this is also visible in the Argand diagram of the susceptibility, cf. Figure 7b.

**Figure 8:**
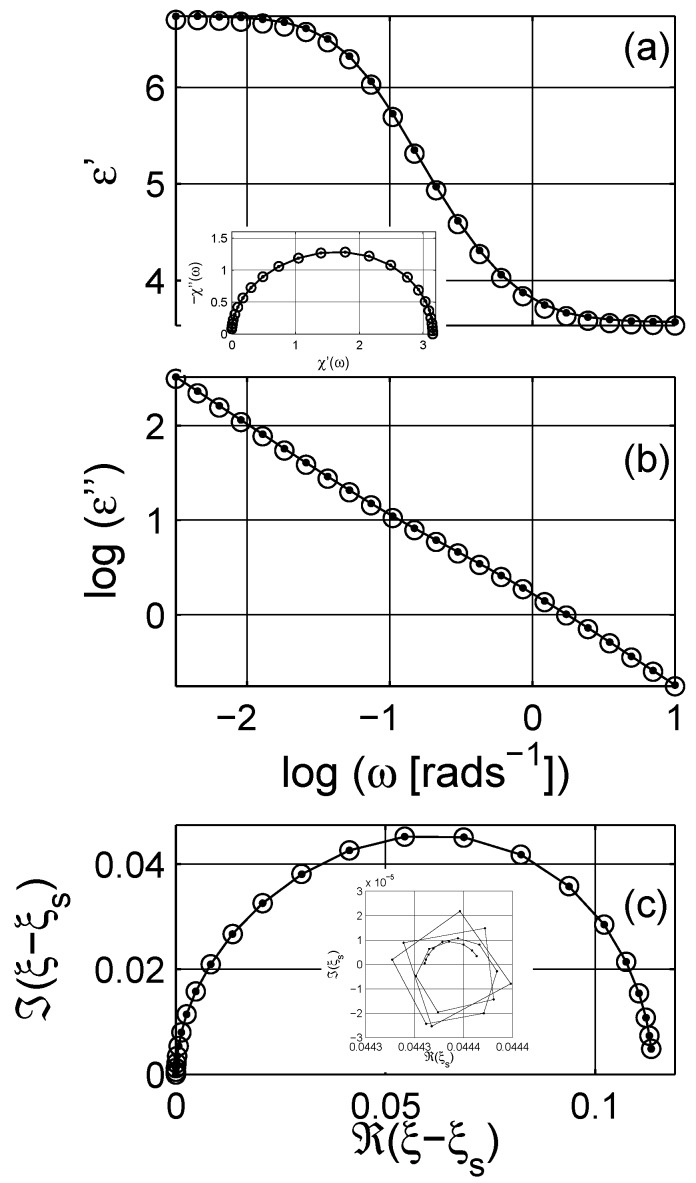
(a) The real and (b) the imaginary parts of the dielectric permittivity calculated with Equation (12) for q=0.3; the permittivities of the matrix and the inclusion phases are $\varepsilon_{m}=2+10^{-12}{\left(ı\varepsilon_{0}\omega \right)}^{-1}$ and $\varepsilon_{i}=10+10^{-10}{\left(ı\varepsilon_{0}\omega \right)}^{-1}$. (c) Argand diagram of scaled permittivity without the percolation strength contribution. The inset in (a) shows the dielectric susceptibility after the subtraction of ohmic conductivity and the permittivity at high frequencies. The inset in (c) shows the Argand plot of $\xi_{s}$ for each point; the values are very narrowly distributed. The lines with points are the simulated data of Equation 12, and the symbols (∘) are the dielectric response calculated with the estimated spectral density function from the proposed numerical algorithm. There is very good agreement between the simulated and the analyzed data sets. Logarithmic scale is base 10.

The reconstructed complex dielectric permittivity and the scaled permittivity are presented in Figure 8. It is important to mention that the fitting is performed at the scaled permittivity level. While numerically calculating the spectral function, the randomly selected spectral parameters *x* are picked between $10^{-3}$ and 1 in logarithmic scale; the pre-distribution is log-linear (for details see [76]). In such an integration limit as in Equation (2), the distribution or the functional contributions of *x*-values lower than $10^{-3}$ are included in the percolation strength $\xi_{s}$.

**Figure 9:**
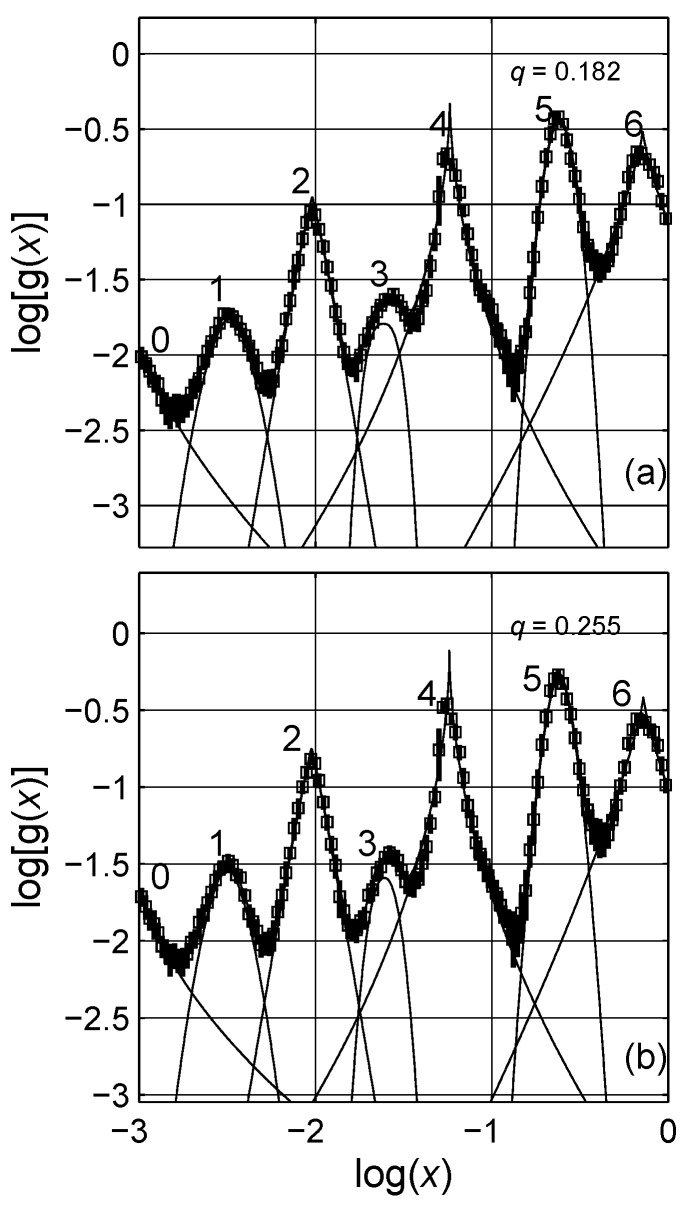
Spectral density functions of the Landau-Lifshitz/Looyenga equation for two concentrations; (a) q=0.2 and (b) q=0.3. The solid lines (——–) are Lévy distributions adapted to the estimated peaks. The numerical data is presented with symbol, error bars. The numbers on the peaks denote the significant peaks. Logarithmic scale is base 10.

**Figure 10:**
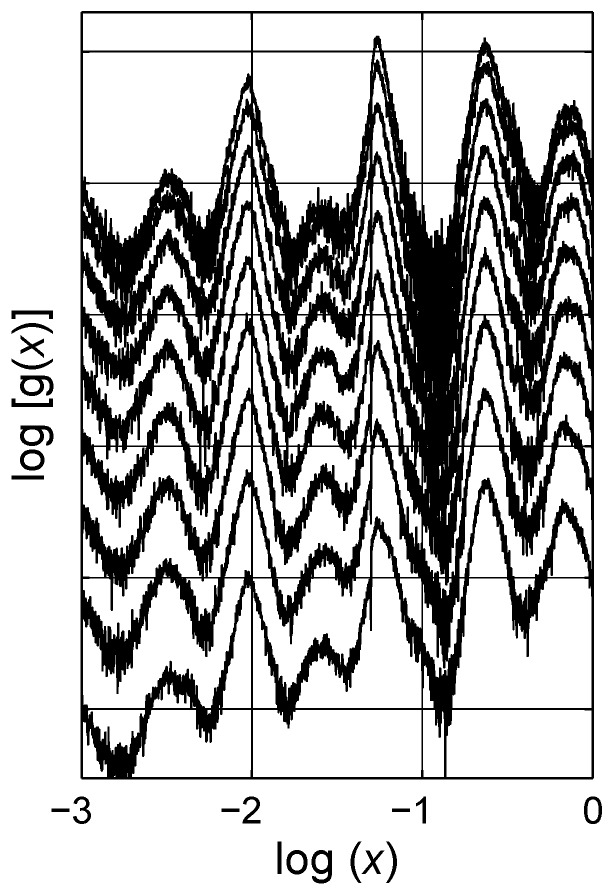
Spectral density functions of the Landau-Lifshitz/Looyenga equation for nine different concentrations, q={0.1,⋯,0.9}. The data are shifted for better comparison. Six peaks are resolved between $x=10^{-3}$ and x=1. Interestingly, the positions of the most probable spectral parameters of the six are not altered with increasing concentration. Logarithmic scale is base 10.

**Figure 11:**
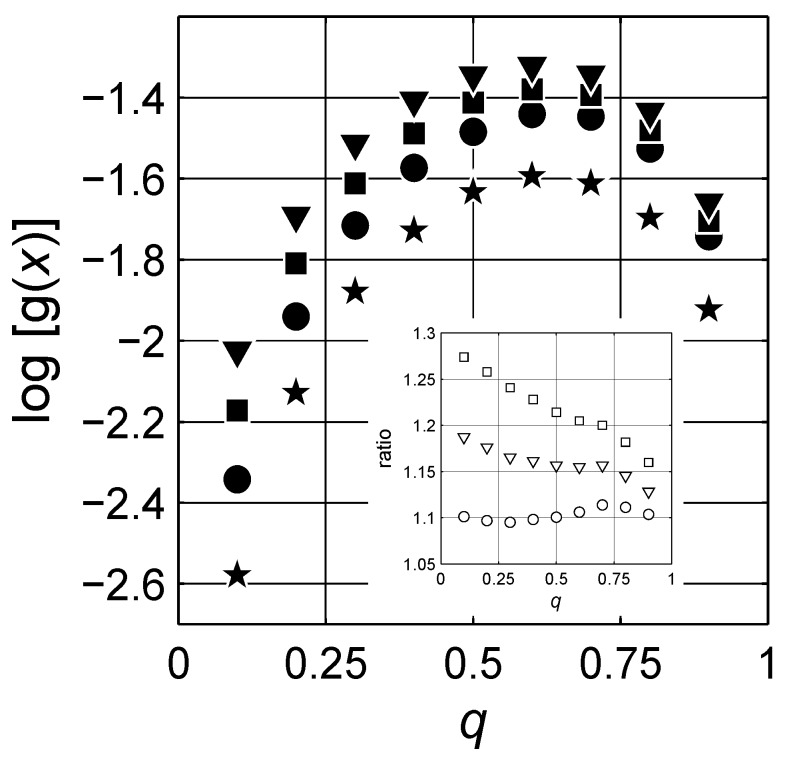
Spectral density function values at constant *x*; log(x)=-2.5 (★), log(x)=-2 (•), log(x)=-1.275 (▾) and log(x)=-0.675 (▪). The inset illustrates the ratios for log{g[log(x)=-2.5]}/log{g[log(x)=-2]} (□), log{g[log(x)=-2.5]}/log{g[log(x)=-1.275]} (▿) and log{g[log(x)=-2.5]}/log{g[log(x)=-0.675]} (∘). Logarithmic scale is base 10.

**Figure 12:**
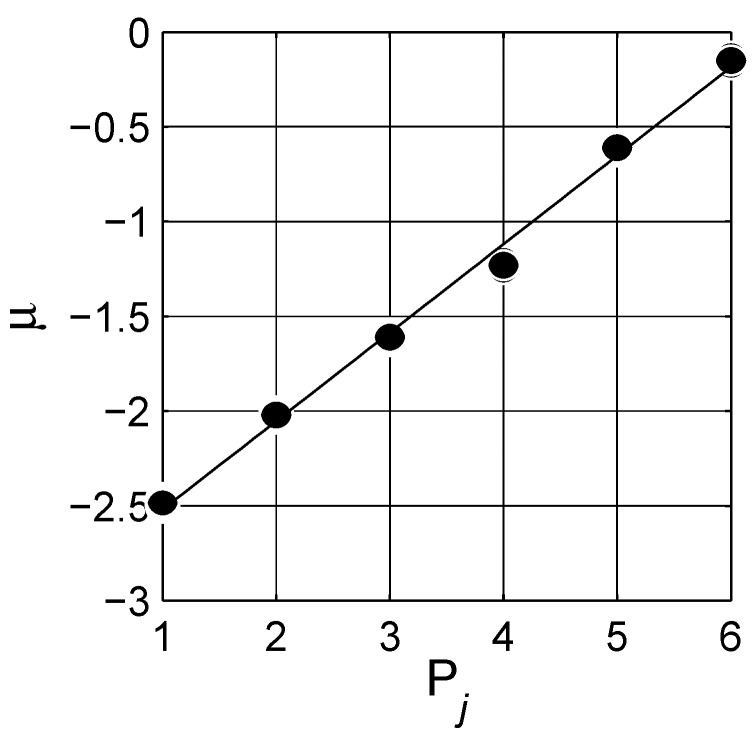
Peak positions, $\mu =\log(\bar{x})$. The solid line is a linear fit with $\mu =0.470P_{j}-2.99$, where *j* is the peak number in Table 1.

The estimated spectral density functions for two concentration levels, q=0.2 and q=0.3, are presented in Figure 9. There are six visible peaks, labeled one to six from left to right. (We do not take peak 0 into account in the analysis.) The estimated integrals of portion of the bell-shaped distributions are presented on the graphs. Each peak is analyzed by the Lévy distribution as mentioned before, and the solid lines show the individual distributions, cf. Figure 9. It is remarkable that the estimated distributions are similar in form but shifted up a bit with increase in concentration *q*. This behavior is an illustration of the self-similar fractal nature of the considered composite system in the LLL expression, in which the topological arrangement does not change significantly with increased concentration; see shifts in SDF of regular lattices with increasing concentrations in Ref. [20]. To support this statement, the spectral density functions of mixtures described with the LLL expression for nine different concentrations are shown in Figure 10. The data are shifted with constant steps in concentration for clarity; however, as observed the spectral function amplitudes do not increase proportionally with increasing inclusion concentration, cf. Table 1 and cf. Figure 11. The difference between peak positions is constant in logarithmic scale $|P_{j}-P_{j-1}|\approx 0.47$, cf. Figure 12.

Table 1 lists not only the positions but also the shape parameters of the Lévy expression for the six peaks resolved for each concentration. The peak positions *μ* are not varied with increasing concentration of the inclusions phase. The amplitude A changes with increasing concentration for each peak. The scale parameter *ζ* and the characteristic exponent *γ* indicate some relation to concentration, but their exact relations are not sought. In Figure 11, we show the change in the amplitude for four of the peaks with increase in the concentration of the inclusions. It is remarkable that around q=0.6 the behaviors of the spectral density functions change; the increase in the amplitude of the peaks with increasing concentration starts to decrease for increasing concentration as q>0.6. The ratio of the three selected peaks to peak 1 are shown in the inset of 11, and a similar activity is also observed in the ratio. Since the ratio between the amplitudes of the selected peaks does not indicate a simple linear relation to concentration *q*, the topological description of the system cannot be qualitatively investigated. However, as mentioned previously, the location and form of the spectral density functions greatly resemble each other, indicating that they are in fact related to the self-similar hierarchical nature of the composites expressed by LLL expression.

**Table 1:** Fit parameters of the Lévy distribution, Equation Equation 13, for the six peaks estimated by the numerical algorithm.

Peak	*q*	A	*μ*	*γ*	*ζ*	Peak	*q*	A	*μ*	*γ*	*ζ*
1	0.1	0.01	-2.47	1.58	6.18	2	0.1	0.06	-2.01	0.89	15.84
	0.2	0.02	-2.49	1.71	6.57		0.2	0.11	-2.02	1.10	12.74
	0.3	0.03	-2.50	1.66	7.29		0.3	0.18	-2.02	1.09	12.83
	0.4	0.04	-2.50	1.75	7.83		0.4	0.22	-2.02	1.20	11.81
	0.5	0.04	-2.50	1.94	7.53		0.5	0.26	-2.03	1.30	11.43
	0.6	0.04	-2.49	2.06	7.81		0.6	0.28	-2.03	1.33	11.46
	0.7	0.04	-2.49	1.98	7.86		0.7	0.28	-2.03	1.22	11.84
	0.8	0.04	-2.48	2.09	7.59		0.8	0.24	-2.03	1.26	11.63
	0.9	0.03	-2.48	1.79	7.20		0.9	0.16	-2.02	1.15	11.54
3	0.1	0.01	-1.62	2.97	8.23	4	0.1	0.23	-1.22	0.58	35.98
	0.2	0.02	-1.62	2.70	8.26		0.2	0.54	-1.24	0.52	49.50
	0.3	0.03	-1.61	2.21	9.34		0.3	0.82	-1.24	0.52	53.04
	0.4	0.03	-1.61	2.27	9.10		0.4	1.04	-1.24	0.56	49.16
	0.5	0.04	-1.61	2.51	8.84		0.5	1.20	-1.24	0.57	48.10
	0.6	0.04	-1.60	2.22	9.05		0.6	1.30	-1.24	0.60	47.57
	0.7	0.03	-1.61	2.58	8.91		0.7	1.26	-1.24	0.60	48.15
	0.8	0.02	-1.61	2.34	8.69		0.8	1.05	-1.24	0.62	45.98
	0.9	0.01	-1.62	2.43	8.32		0.9	0.58	-1.24	0.62	41.94
5	0.1	0.20	-0.62	1.85	10.89	6	0.1	0.22	-0.16	0.67	15.10
	0.2	0.36	-0.62	1.90	10.51		0.2	0.31	-0.14	0.77	10.98
	0.3	0.54	-0.62	1.70	11.15		0.3	0.38	-0.14	0.81	10.58
	0.4	0.62	-0.62	1.72	10.89		0.4	0.40	-0.14	0.80	9.84
	0.5	0.69	-0.61	1.58	10.90		0.5	0.37	-0.14	0.97	9.08
	0.6	0.65	-0.61	1.63	10.25		0.6	0.33	-0.14	1.02	8.87
	0.7	0.56	-0.61	1.66	9.57		0.7	0.28	-0.14	1.08	8.93
	0.8	0.39	-0.60	1.80	8.46		0.8	0.18	-0.14	1.33	8.22
	0.9	0.26	-0.60	1.50	9.33		0.9	0.10	-0.14	1.34	8.45

**Figure 13:**
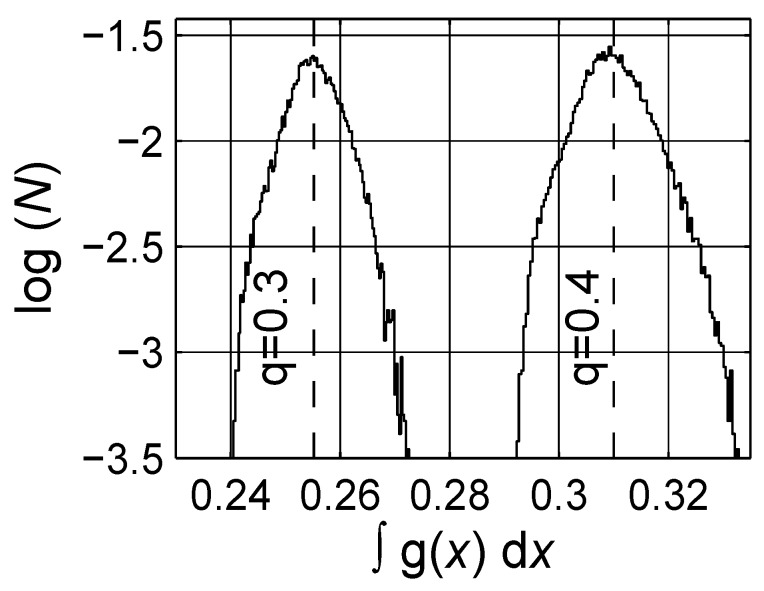
The number density distribution of estimated $\int g\left(x\right)dx\equiv \bar{q}$ for two concentration *q* levels, q={0.3,0.4}. The dashed vertical lines are the expectation values of $\bar{q}$, which are 0.255±0.065 and 0.310±0.096 for concentration levels 0.3 and 0.4, respectively. Logarithmic scale is base 10.

**Figure 14:**
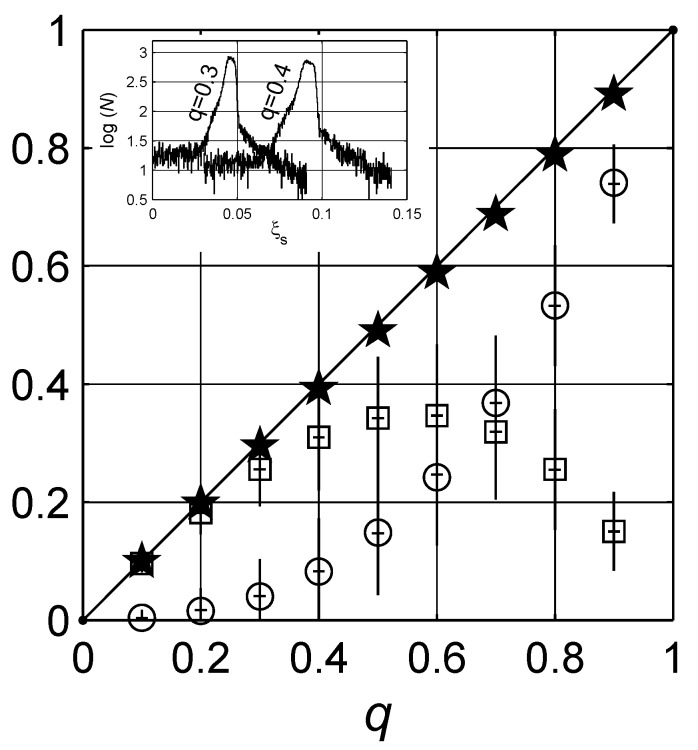
The expected values of constants, $\bar{q}$ and $\bar{\xi_{s}}$, in the spectral density approach. The integral of g(x) yields the concentration of the inclusions $\bar{q}$, denoted by open squares (□). The expected value of percolation strength is $\bar{\xi }$ denoted by open circles (∘). Both values are also presented with error bars. Addition of the two expected values $\bar{\xi_{s}}+\bar{q}$, denoted by stars (★), leads to the actual concentration *q* as in Equation (20), which is denoted by the solid line (——–). In the inset, the number distributions of $\xi_{s}$, percolation strength, estimated in each Monte Carlo cycle for q=0.3 and q=0.4 are illustrated with the most expected $\bar{\xi_{s}}$ being 0.041±0.006 and 0.083±0.015, respectively, for the two concentrations.

Finally, the statistical analysis of the concentration calculated from the integral of the spectral density function (integral expression on the right-hand side of Equation 2) and the value of the percolation strength $\xi_{s}$ at each Monte Carlo step are summarized in Figure 13 and Figure 14. The number distribution of the integral of the spectral density function calculated at each Monte Carlo cycle is not centered at the actual concentration *q* taken but deviated. The deviation indicates that there is a percolation path network structure, cf. Figure 13. As expected from the definition of the spectral density function, Equation (20), addition of the estimated concentration $\bar{q}$ and percolation strength $\bar{\xi_{s}}$ yield numerical values very close to the actual concentration *q*. The value $\bar{q}$ is calculated from the distribution. It is striking that the applied numerical method is capable of estimating the concentration of the filler material when it is not known in advance.

## 6. Application of the Havriliak-Negami Expression

Previously, it has been stated that the scaled permittivity *ξ* in Equation (3) can be expressed as in the conventional form as in the case of dielectric relaxation [21], Equation (5). To illustrate and verify this statement, we apply a complex nonlinear least-squares curve fit algorithm to the scaled permittivity of the LLL expression, denoted as $\xi^{\mathrm{LLL}}$ below. The error in the curve fitting procedure is used to quantify the fitness of the model function in Equation (5). The error is calculated as the sum of the relative error at each point as follows,

(14) $$\begin{array}{c} E=\sum {\left[\frac{ℜ\left(\xi^{\mathrm{LLL}}\right)-ℜ\left(\xi^{\mathrm{HN}}\right)}{ℜ(\xi^{\mathrm{LLL}})}\right]}^{2}+{\left[\frac{ℑ\left(\xi^{\mathrm{LLL}}\right)-ℑ\left(\xi^{\mathrm{HN}}\right)}{ℑ(\xi^{\mathrm{LLL}})}\right]}^{2} \end{array}$$

Here $\xi^{\mathrm{HN}}$ is the model expression of Equation (5). The fit results are listed in Table 2, where the model values for the spectral parameter *x*, concentration *q* and percolation strength $\xi_{s}$ are presented with over-lines as in the previous section.

**Table 2:** Fit parameters of the Havriliak-Negami [90] expression to scaled permittivity, Equation (5). The error E is calculated with Equation (14).

*q*	*α*	*β*	$\bar{x}$	$\bar{q}$	$\bar{\xi_{s}}$	E
0.1	1.034	0.438	0.536	0.095	0.000	1.19$\times 10^{-3}$
0.2	0.822	0.555	0.514	0.200	0.009	7.28$\times 10^{-6}$
0.3	0.716	0.635	0.464	0.297	0.032	2.45$\times 10^{-4}$
0.4	0.850	0.454	0.522	0.345	0.066	4.27$\times 10^{-6}$
0.5	0.800	0.470	0.494	0.391	0.128	2.06$\times 10^{-5}$
0.6	0.828	0.422	0.493	0.397	0.216	4.08$\times 10^{-6}$
0.7	0.822	0.409	0.475	0.371	0.342	5.20$\times 10^{-6}$
0.8	0.815	0.399	0.456	0.300	0.510	6.41$\times 10^{-6}$
0.9	0.810	0.390	0.438	0.179	0.727	7.60$\times 10^{-6}$

**Figure 15:**
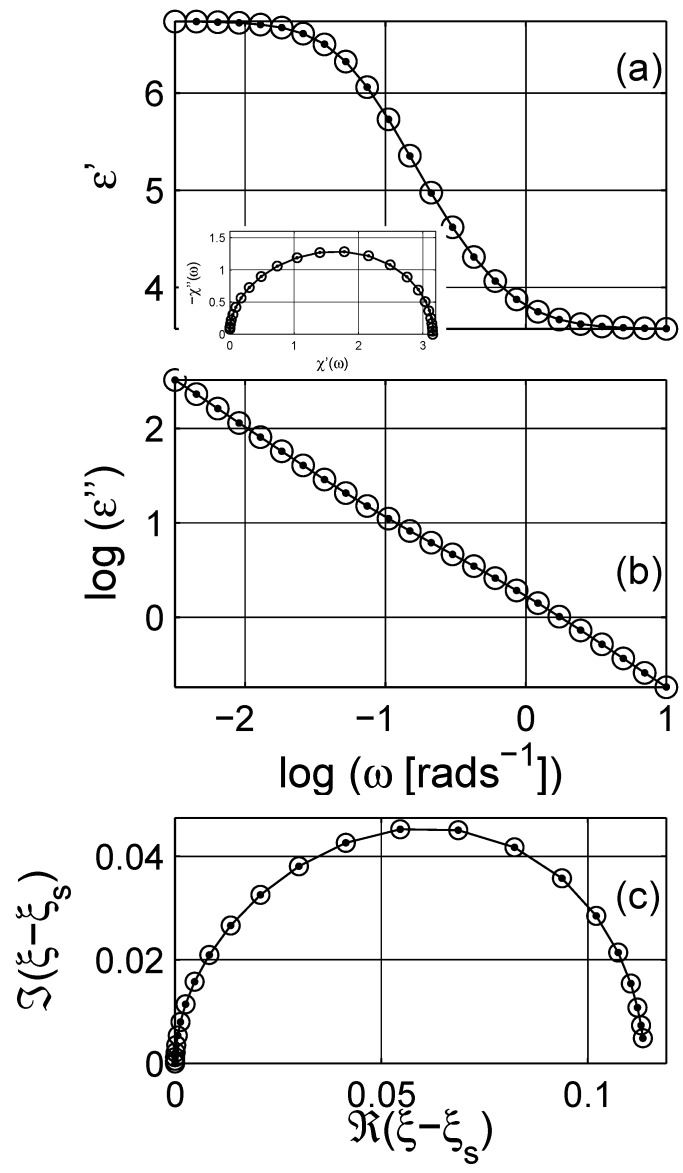
(a) The real and (b) the imaginary parts of the dielectric permittivity calculated with Equation (12) for q=0.3 and the modeled response obtained with the application of the Havriliak-Negami expression to the scaled permittivity. The permittivities of the matrix and the inclusion phases are the same as in Figure 8. (c) Argand diagram of scaled permittivity without the percolation strength contribution. The inset in (a) shows the dielectric susceptibility after the substruction of ohmic conductivity and the permittivity at high frequencies. The lines with points are the simulated data, and the symbols (∘) are the data estimates of the Havriliak-Negami expression, Equation 5. There is very good agreement between the simulated and the analyzed data sets. Logarithmic scale is base 10.

**Figure 16:**
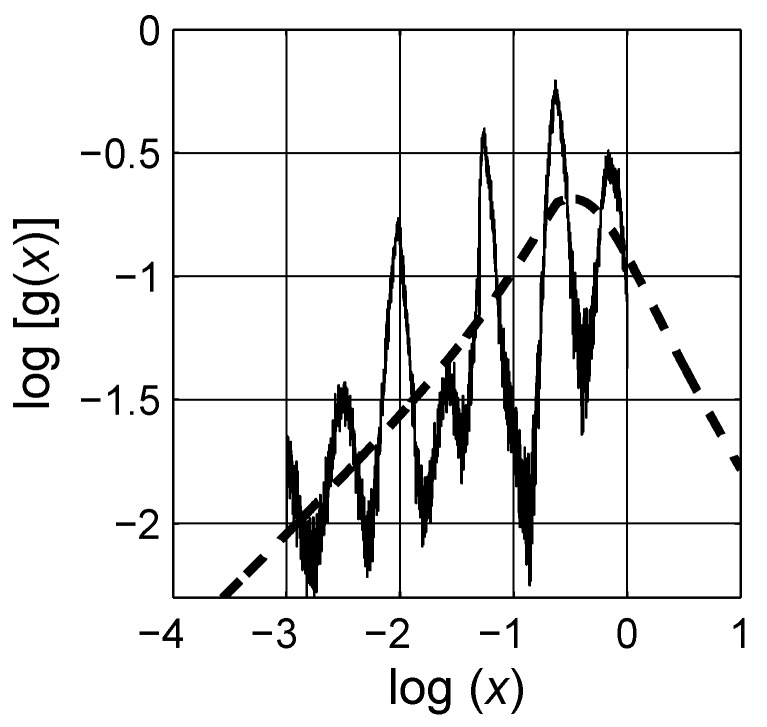
Comparison of the distributions sfg obtained by the Havriliak-Negami approach (– – –) and the novel numerical approach (——–). Observe that the Havriliak-Negami distribution also considers spectral parameter *x* values larger than 1, log(x)>0. Logarithmic scale is base 10.

**Figure 17:**
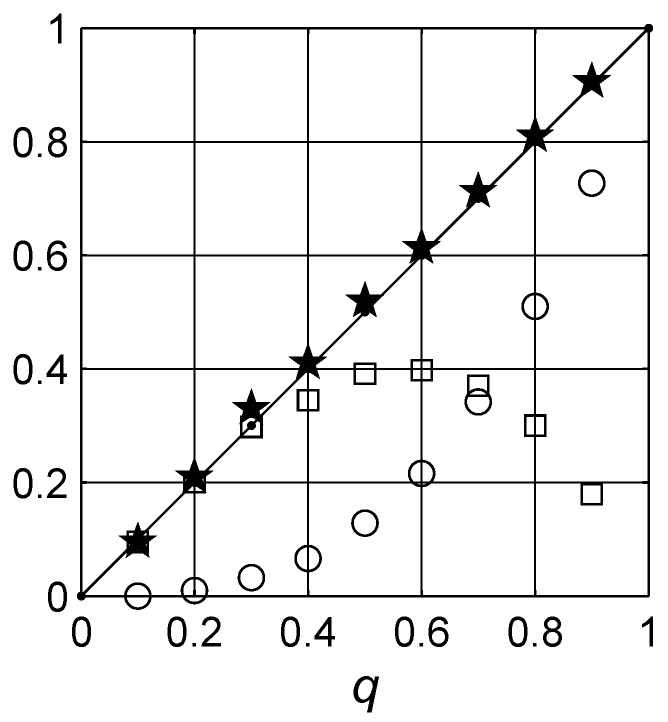
The most probable values of constants $\bar{q}$ and $\bar{\xi_{s}}$ from application of the Havriliak-Negami expression. The integral of g(x) yields the concentration of the inclusions $\bar{q}$, denoted by open squares (□). The percolation strength is $\bar{\xi }$, denoted by open circles (∘). The sum of the two constants, $\bar{\xi_{s}}+\bar{q}$, denoted by stars (★), leads to the actual concentration, which is denoted with the solid line (——–).

The fit results are shown in Figure 15 for the q=0.3 case in a form similar to Figure 8. There are actually no particular differences between the two methods, except that the numerical techniques based on the Monte Carlo algorithm are capable of resolving individual peaks, as shown in the comparison graph in Figure 16. It is not clear, for example, from the Havriliak-Negami approach that the system indicates a self-similar fractal-like structure. One should note that the Havriliak-Negami distribution, cf. § 7. [73], presented below, was spread evenly over spectral parameter values larger than one, x>1, which is not possible in the spectral density representation. However, when analyzing the data it is very convenient and trivial to implement available curve fitting programs. Similar to Figure 14, the estimated concentration $\bar{q}$ and percolation strength $\bar{\xi_{s}}$ from the parametric analysis satisfy the condition in Equation 20 as shown in Figure 17.

## 7. Conclusions

In this review, we first presented the significance of the spectral density representation and then derived an expression for dielectric mixtures that resembles the distribution of relaxation times representation in dielectric relaxation phenomenon. In the derivation we used the spectral density representation of Ghosh and Fuchs [33]. It is shown that existing knowledge on dielectric relaxation theory can be applied to dielectric properties of composites. To confirm the hypothesis, both a similar method for estimating the distribution of relaxation times and an extensively used empirical formula to express dielectric relaxation are employed to estimate the spectral density functions of composites simulated with the LLL expression. The numerical method, based on the Monte Carlo technique, estimated a couple of peaks that did not change their location in the spectra with increased concentration of inclusions. This static behavior of the spectra indicates that there exists a hierarchical structural order in the composite. As a result, we infer that the LLL expression is proper for systems with a self-similar fractal nature, such as composites with colloid aggregates and porous materials. We have explicitly shown why the LLL expression could be applied to describe the dielectric properties of powdered and porous systems. Last but not least, the findings are significant to confirm the structure of composite systems, whose dielectric permittivities are described in the literature with the LLL expression.

## Appendix A. Derivation of the Simple Form

Equation (1) is expanded as follows

(15) $$\begin{array}{c} \frac{\varepsilon_{e}-\varepsilon_{m}}{\varepsilon_{m}}=q\;A\left(\frac{\varepsilon_{e}-\varepsilon_{m}}{\varepsilon_{m}}\right)+q\int_{0}^{1}\frac{G(x)dx}{{\left(\frac{\varepsilon_{e}-\varepsilon_{m}}{\varepsilon_{m}}\right)}^{-1}+x} \end{array}$$

Let $\varepsilon_{i}-\varepsilon_{j}\equiv \Delta_{ij}$ then

(16) $$\begin{array}{c} \frac{\Delta_{\mathrm{em}}}{\varepsilon_{m}}=q\;A\frac{\Delta_{\mathrm{im}}}{\varepsilon_{m}}+\int_{0}^{1}\frac{qG\left(x\right)\Delta_{\mathrm{im}}dx}{\varepsilon_{m}+\Delta_{\mathrm{im}}x} \end{array}$$

Now, multiply both sides by $\varepsilon_{m}$ to obtain

(17) $$\begin{array}{c} \Delta_{\mathrm{em}}=q\;A\Delta_{\mathrm{im}}+\int_{0}^{1}\frac{qG\left(x\right)\Delta_{\mathrm{im}}dx}{1+\varepsilon_{m}^{-1}\Delta_{\mathrm{im}}x} \end{array}$$

Finally, let $\Delta_{\mathrm{em}}/\Delta_{\mathrm{im}}\equiv \xi$ and $qA\equiv \xi_{s}$, and we obtain Equation (2). The properties of G and *A* are such that [33,78,113]

(18) $$\begin{array}{ccc} A+\int_{0^{+}}^{1}G\left(x\right)dx & = & 1\; \end{array}$$

(19) $$\begin{array}{ccc} \int_{0^{+}}^{1}xG\left(x\right)dx & = & \frac{q(1-q)}{d}\; \end{array}$$

Here, *d* is the dimension of the system. When we consider our new notation, then

(20) $$\begin{array}{ccc} \xi_{s}+\int_{0^{+}}^{1}g\left(x\right)dx & = & q \end{array}$$

(21) $$\begin{array}{ccc} \int_{0^{+}}^{1}xg\left(x\right)dx & = & \frac{\left(1-q\right)}{d} \end{array}$$

## Appendix B. Havriliak-Negami Distribution Function

Havriliak and Negami [90] have combined the works of Cole and Cole [93] and Davidson and Cole [92] and have expressed the dielectric dispersion with an asymmetric formula as presented in Equation (5). When α=β=1, Equation (5) becomes the simple Debye and Maxwell Garnett equations for dielectrics or dielectric mixtures, respectively. Other interesting cases are the when α=1; Davidson-Cole expression and the when β=1; Cole-Cole expression. Havriliak and Negami [114] have used the distribution of relaxation times as expressed by Davidson and Cole [92] and have substituted sexp(±ıπ) for ıs. The solution for the distribution relaxation times and the spectral density function g(s), then, becomes

(22) $$g\left(s\right)=\frac{1}{\pi }\left|\frac{10^{s\alpha \beta }\sin\left(\beta \Theta \right)}{{\left[10^{2s\alpha }+2\;\cdot \;10^{s\alpha }\cos\left(\alpha \pi \right)+1\right]}^{\beta /2}}\right|$$

where

$$\Theta =\arctan\left[\frac{\sin(\alpha \pi )}{10^{s\alpha }+\cos\left(\alpha \pi \right)}\right]$$

and $s=\log(x/\bar{x})$, with $\bar{x}$ being the most probable spectral parameter.
